# Use of intraoperative right ventricular strain as a predictor of outcomes in adult heart transplant patients

**DOI:** 10.3389/fcvm.2026.1890033

**Published:** 2026-10-07

**Authors:** John Bryant, Becky Settlemire, Awab Ahmad, Robert Deegan, Drexel A. Neumann, Bantayehu Sileshi, Aaron Williams, Swaroop Bommareddi, John Trahanas, Kelly Schlendorf, Mark Petrovic, Chen Chia Wang, Afif Kraitem, Neal Duggal, Taylor Ziegler, Amit Jhaveri, Michael Baker, Mias Pretorius, Ashish Shah, Christina Anne Jelly, Brian Lima

**Affiliations:** 1 Department of Anesthesiology, Vanderbilt University Medical Center, Nashville, TN, United States; 2 Echocardiography Laboratory, Vanderbilt University Medical Center, Nashville, TN, United States; 3 Department of Cardiac Surgery, Vanderbilt University Medical Center, Nashville, TN, United States; 4 Division of Cardiovascular Medicine, Department of Medicine, Vanderbilt University Medical Center, Nashville, TN, United States; 5 Vanderbilt University School of Medicine, Nashville, TN, United States; 6 Department of Anesthesiology, University of Michigan, Ann Arbor, MI, United States

**Keywords:** heart transplantation, primary graft dysfunction, right-ventricular function, right-ventricular strain, speckle-tracking echocardiography, transesophageal echocardiography

## Abstract

**Background:**

Right ventricle (RV) evaluation in the perioperative period is challenging for anesthesiologists, given the limited objective assessment methods available for transesophageal echocardiography (TEE). Speckle-tracking strain imaging may offer a more objective evaluation of RV function. This study evaluated the predictive value of intraoperative RV free wall strain (RVFWS) for early outcomes following orthotopic heart transplantation (OHT).

**Methods:**

This was a single-center retrospective cohort study of 147 adults who underwent isolated OHT between January 2025 and December 2025. Patients with multiorgan transplant, congenital cases, and those without measurable intraoperative RVFWS were excluded. Post-bypass RVFWS was measured using TEE at a single time point with speckle-tracking analysis. The primary outcome was 90-day mortality. Secondary outcomes included severe primary graft dysfunction, 30-day mortality, need for postoperative venoarterial extracorporeal membrane oxygenation and continuous renal replacement therapy (CRRT), vasoactive-inotropic score, and cardiac index at 24 and 72 h, and intensive care unit length of stay. Multivariable Firth-penalized logistic and median quantile regressions were used, adjusted for *a priori* pre-transplant weighted risk score.

**Results:**

The median post-bypass RVFWS was −13.9% IQR (interquartile range) −19.3 to −9.6. Fourteen recipients (9.5%) died within 90 days of transplantation. After adjusting for the pre-transplant risk score, RVFWS was independently associated with higher 90-day mortality [odds ratio (OR) 2.84 per 5% decrease in magnitude, 95% confidence interval 1.65–5.58; *p* < 0.001]. Among the secondary outcomes, the associations with severe primary graft dysfunction (OR 2.70; raw *p* = 0.001), CRRT (OR 1.58; raw *p* = 0.001), and 30-day mortality (OR 2.94; raw *p* = 0.002) remained statistically significant after Bonferroni adjustment for eleven secondary comparisons (adjusted *p* = 0.011, 0.011, and 0.022, respectively). RVFWS demonstrated higher discrimination for 90-day mortality [area under the curve (AUC) 0.83] than qualitative intraoperative RV grading (AUC 0.68), although this difference in AUC did not reach statistical significance. RVFWS retained independent prognostic value in a combined model.

**Conclusions:**

Intraoperative post-bypass RVFWS is a feasible and objective intraoperative measure that is independently associated with early adverse outcomes after adult OHT. RVFWS shows promise as a signal of early allograft dysfunction that is not fully explained by pre-transplant risk factors.

## 1 Introduction

The right ventricle (RV) plays a crucial role in the overall cardiac performance. It differs from the left ventricle (LV) in geometry, wall thickness, and contraction mechanics, making it difficult to extrapolate conventional LV-based assessment tools to the RV (1). The assessment of RV function in heart transplant recipients remains a clinical challenge. Echocardiography is the first-line tool for assessing RV function; however, no single parameter is universally accepted as ideal, and clinicians frequently rely on subjective visual assessment (2, 3). Quantitative measurements, including RV fractional area change (RV FAC), tricuspid annular plane systolic excursion (TAPSE), tissue Doppler (TDI) S’ velocity, and the RV myocardial performance index, have shown variable correlations with RV function when compared with cardiac magnetic resonance imaging (4).

Strain imaging has emerged as a promising tool for quantifying myocardial function and contractility. Strain reflects myocardial deformation and can be assessed using tissue Doppler or, more commonly, speckle-tracking echocardiography, which tracks myocardial motion frame by frame over the cardiac cycle (5). Strain offers several potential benefits over other measures of myocardial function, including the early detection of dysfunction, identification of regional differences, and quantification of changes in the timing of myocardial contraction (6, 7). With regard to the RV, strain can be measured as RV global longitudinal strain (RVGLS) or RV free wall strain (RVFWS). RVGLS averages the strain across the RV, while RVFWS measures only the free wall, excluding the septum (8). Strain is reported as a negative value, and a *more negative* value (i.e., greater absolute magnitude) indicates better contractile function. Normal values vary by patient population and the method of measurement. However, in general, RVGLS values between −20% and −25% and RVFWS values between −25% and −30% are considered normal; values closer to zero (smaller in absolute magnitude) indicate worsening function (9).

In heart transplant recipients, RV strain has been associated with rejection, morbidity, and mortality in studies performed weeks to months after transplantation. Although these studies are promising, it remains unclear whether *perioperative* RV strain provides additional prognostic information or better reflects early allograft performance (10, 11). Intraoperative RV strain measured by transesophageal echocardiography (TEE) has been assessed in other cardiac surgery populations, including those undergoing left-ventricular assist device (LVAD) implantation and mitral valve surgery; however, evidence in heart transplant recipients is limited (12, 13).

The aim of this study was to evaluate intraoperative RV strain measurements during orthotopic heart transplantation (OHT) as an indicator of postoperative allograft performance. *We primarily hypothesized that a lower-magnitude (less negative) post-bypass RVFWS would be associated with higher 90-day mortality after heart transplantation.* Second, we hypothesized that lower-magnitude post-bypass RVFWS would be associated with worse early postoperative outcomes [30-day mortality, need for postoperative venoarterial extracorporeal membrane oxygenation (VA-ECMO), need for continuous renal replacement therapy (CRRT), higher vasoactive-inotropic score (VIS), and longer intensive care unit (ICU) length of stay], and that RVFWS would provide additional prognostic information beyond qualitative intraoperative RV assessment.

## 2 Materials and methods

### 2.1 Study design and population

This single-center, retrospective cohort study was conducted at the Vanderbilt University Medical Center (VUMC), a tertiary academic transplant center. All adult heart transplant recipients between January 2025 and December 2025 were considered for inclusion. Of the 195 recipients identified during the study period, 7 were excluded for missing intraoperative RVFWS measurements, 15 for congenital heart disease etiology, and 26 for multiorgan transplantation, leaving 147 patients for analysis (Figure 1). Of the 147 included recipients, 76 (51.7%) received hearts from donors after brain death (DBD) and 71 (48.3%) from donors after circulatory death (DCD), consistent with our institutional DCD heart-transplant program. Patients requiring intraoperative VA-ECMO or temporary mechanical circulatory support were not pre-emptively excluded; only patients for whom a post-bypass RVFWS image could not be technically acquired or adequately analyzed were excluded for “missing RVFWS.”

**Figure 1:**
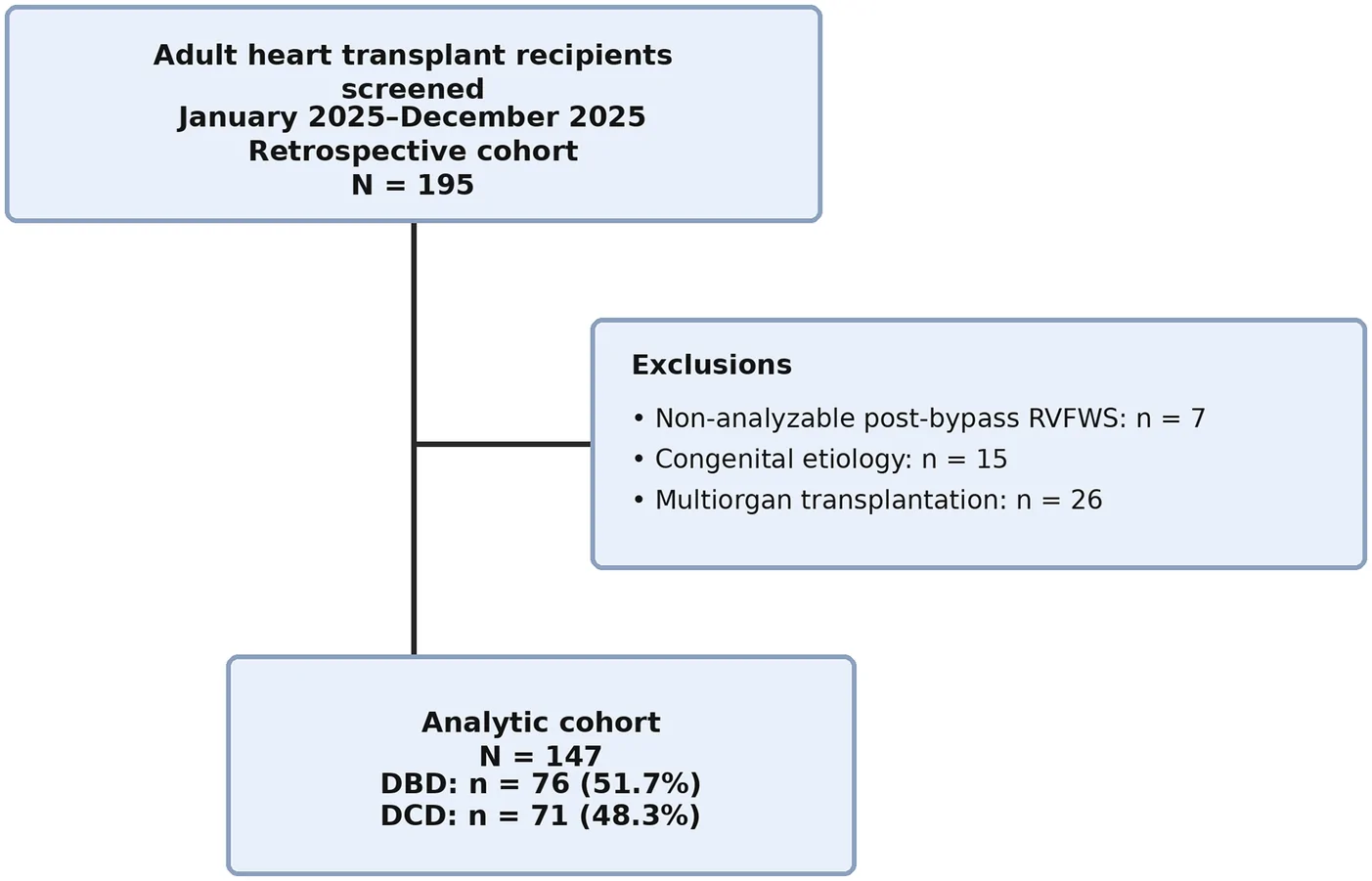
Study population selection. RVFWS, right-ventricular free wall strain.

The study was approved by the VUMC Institutional Review Board (IRB protocol #241909), and the requirement for individual patient consent was waived because of the retrospective nature of the analysis.

The surgical implantation technique varied across the cohort and was not standardized for this analysis. Perioperative inotropic support was initiated and titrated at the discretion of the attending cardiac anesthesiologist and attending cardiac surgeon, and typically included combinations of epinephrine, milrinone, dopamine, and dobutamine. Inhaled epoprostenol is the routine inhaled pulmonary vasodilator used for postoperative pulmonary hypertension and right-ventricular afterload management at our institution.

### 2.2 Intraoperative RV strain (exposure)

The primary exposure was post-bypass RVFWS, measured on TEE after separation from cardiopulmonary bypass and prior to transport to the ICU. Imaging was performed using Philips Epiq CVx ultrasound systems with X8-2t TEE probes (Philips, Andover, MA, USA). Post-bypass strain was measured offline by a single observer using TomTec Image Arena (TomTec Imaging Systems, Unterschleißheim, Germany) from an RV-focused, mid-esophageal four-chamber view. RVGLS was not measured because of challenging post-transplant imaging windows and the dependence of RVGLS on LV mechanics. The RVFWS values analyzed here were quantified offline after completion of the case and were not available to the intraoperative team. In a subset of patients (*n* = 66), an attending anesthesiologist independently recorded a real-time strain measurement for the observer-agreement analysis; intraoperative management, including the decision to initiate VA-ECMO, was based on the team's real-time qualitative TEE assessment and hemodynamics. Residual influence of these real-time measurements on clinical decisions in that subset cannot be entirely excluded.

To minimize the variability introduced by ongoing intraoperative resuscitation, the RVFWS measurement was acquired at the end of the case after chest closure, when the final inotropic support and volume status were intended to be at a stabilized end-of-case state. Specifically, the last adequate RV-focused mid-esophageal four-chamber image acquired before transport to the ICU was selected for each patient—operationalized as the final loop in which the RV free wall, apex, and tricuspid annulus were all clearly visualized with minimal foreshortening or dropout. Patients in whom no adequate end-of-case image could be obtained were excluded as “missing RVFWS” (*n* = 7; see Figure 1). For the subset of patients who required intraoperative VA-ECMO prior to transport to the ICU (*n* = 9; 6.1%), RVFWS was acquired prior to ECMO cannulation, so that all measured strain values in this analysis reflected intrinsic post-bypass RV function that was not unloaded by mechanical circulatory support.

Inter- and intra-observer variability were assessed as follows. To evaluate inter-observer variability, post-processing RV strain measurements by the study observer were compared with intraoperative real-time measurements performed independently by a separate attending anesthesiologist in 66 patients. To evaluate intra-observer variability, RV-focused post-bypass images from 20 different patients were independently re-measured by the same study observer on a separate occasion, who was blinded to the original measurement.

### 2.3 Qualitative intraoperative RV assessment

In addition to the quantitative RVFWS, the qualitative intraoperative RV function was graded by the attending cardiac anesthesiologist at the time of separation from cardiopulmonary bypass using a four-point ordinal scale (none, mild, moderate, severe). Grading was based on standard intraoperative TEE assessment, incorporating RV size, free wall motion, septal motion, and tricuspid regurgitation severity. Because only two recipients were graded as severe, the moderate and severe categories were combined for analysis.

Qualitative grading was performed by the attending cardiac anesthesiologist at the time of surgery and documented in the operative TEE report. Inter- and intra-observer variability for qualitative grading was not formally assessed in this study, which is addressed as a limitation in the Discussion section.

### 2.4 Outcomes

The primary outcome was 90-day all-cause mortality. The 90-day mortality was selected as the primary endpoint because it captures the most clinically definitive consequence of early graft dysfunction and is a standard, well-adjudicated outcome in the heart-transplant literature.

The pre-specified secondary outcomes included severe primary graft dysfunction (PGD); 30-day mortality; need for postoperative VA-ECMO—categorized as initiated before transfer from the operating room (“VA-ECMO before ICU”) versus initiated after arrival in the ICU (“VA-ECMO after ICU”) in accordance with how this is documented in our institutional registry; need for postoperative CRRT; ICU length of stay; VIS at 24 and 72 h; cardiac index at 24 and 72 h; and intra-aortic balloon pump (IABP) use at 24 h.

Severe PGD was defined by adapting the 2014 International Society for Heart and Lung Transplantation (ISHLT) consensus criteria, as left-ventricular, right-ventricular, or biventricular graft dysfunction occurring within 24 h of transplantation and requiring either high-dose inotropic support (left-ventricular ejection fraction ≤40% or hemodynamic compromise with right-atrial pressure >15 mmHg, pulmonary capillary wedge pressure >20 mmHg, cardiac index <2.0 L/min/m^2^, and inotrope score >10), or mechanical circulatory support (VA-ECMO, intra-aortic balloon pump, or temporary ventricular assist device) (29). This composite is broader than the 2014 consensus category of severe PGD-LV, which excludes patients supported with high-dose inotropes or an intra-aortic balloon pump alone. PGD events in this cohort included isolated right-ventricular, isolated left-ventricular, and biventricular PGD. Categorical subtype-specific analyses were not performed because of the small subtype event counts.

The VIS was calculated using the following standard formula: dopamine (μg/kg/min) + dobutamine (μg/kg/min) + 100 × epinephrine (μg/kg/min) + 100 × norepinephrine (μg/kg/min) + 10 × milrinone (μg/kg/min) + 10,000 × vasopressin (units/kg/min) + 100 × phenylephrine (μg/kg/min). The cardiac index was calculated from continuous thermodilution pulmonary artery catheter measurements (cardiac output divided by body surface area, expressed in L/min/m^2^) at 24 and 72 h postoperatively.

### 2.5 Statistical analysis

Pre-transplant donor and recipient characteristics and post-transplant outcomes were summarized as mean (standard deviation) or median (interquartile range, IQR) for continuous variables and as counts (percentages) for categorical variables. All analyses were performed using R version 4.4.1 (R Foundation for Statistical Computing, Vienna, Austria).

#### 2.5.1 Association of RVFWS with outcomes

Multivariable Firth-penalized logistic regression was used to assess the independent association between RVFWS and the binary outcomes. The Firth correction was chosen, given the limited sample size and low event rates, to reduce small-sample bias and address potential separation. For continuous outcomes, median (quantile) regression (*τ* = 0.5) was used to estimate the median change associated with the RVFWS. Effect estimates were reported as odds ratios (ORs) for binary outcomes and median change estimates for continuous outcomes, each with 95% confidence intervals (CIs).

To limit overfitting given the limited sample size and event rate, a pre-transplant weighted risk score was constructed *a priori* based on donor and recipient characteristics consistently associated with graft failure and post-transplant mortality in the heart transplant literature (Supplementary Table S1). These characteristics included donor age (14–16), donor–recipient sex matching (17), diabetic donors (18), donor-predicted heart mass ratio (19), allograft ischemic time (20–22), prior LVAD, and prior sternotomy. Multivariable models were adjusted for this risk score to limit overfitting while accounting for the baseline risk.

The risk score was developed in a larger institutional heart-transplant dataset using ridge regression, with severe primary graft dysfunction and/or early mortality as the outcome. The regression coefficients were exponentiated and proportionally weighted to generate an additive score reflecting the relative contribution of each predictor. The score was developed only for multivariable adjustment in this study and was not intended to serve as an externally validated clinical decision-making tool. This score was derived *a priori* using our institutional heart-transplant dataset through December 2024 (*n* ≈ 600), entirely prior to and non-overlapping with the present analytic cohort (transplanted January–December 2025); therefore, adjustment for the score does not constitute a circular adjustment. Full derivation details, including the exponentiation of ridge-regression coefficients (e*^β^*) into proportionally rescaled additive point values, are provided in the legend of Supplementary Table S1.

##### 2.5.1.1 Multiple-comparisons adjustment

To address the risk of inflated type I error across the 11 pre-specified secondary outcomes, both Bonferroni-adjusted and Benjamini–Hochberg (BH) false-discovery-rate (FDR) *q*-values were calculated alongside raw *p*-values and are reported in Table 1. The primary outcome (90-day mortality) was tested at *α* = 0.05 without multiple-comparisons adjustment, consistent with the hierarchical testing convention. For secondary outcomes, a Bonferroni-adjusted threshold of *p* < 0.05/11 ≈ 0.0045 was used as the most conservative criterion, and a BH-FDR threshold of *q* < 0.05 was used as the less-conservative criterion appropriate for an exploratory cohort study with biologically related outcomes.

**Table 1:** Independent association of post-bypass RVFWS with postoperative outcomes after adjustment for the pre-transplant weighted risk score.

Outcome	OR (95% CI)/median change (95% CI)	Raw *p*	Bonferroni *p*^a^	BH-FDR *q*^b^
Primary outcome				
90-day mortality	2.84 (1.65 to 5.58)	**<0**.**001**	—	—
Secondary outcomes				
Severe PGD^c^	2.70 (1.47 to 5.86)	**0**.**001**	**0**.**011**	**0**.**006**
Post-transplant CRRT	1.58 (1.18 to 2.16)	**0**.**001**	**0**.**011**	**0**.**006**
30-day mortality	2.94 (1.42 to 8.01)	**0**.**002**	**0**.**022**	**0**.**007**
VIS at 24 h	+0.76 (0.00 to +1.28)	**0**.**01**	0.110	**0**.**028**
VA-ECMO before ICU	1.71 (1.00 to 3.21)	0.04	0.440	0.088
VA-ECMO after ICU	1.63 (0.91 to 3.27)	0.10	1.000	0.183
Cardiac index at 72 h	0.04 (−0.07 to +0.08)	0.31	1.000	0.481
ICU length of stay, days	+0.40 (−0.20 to +1.34)	0.35	1.000	0.481
VIS at 72 h	−0.19 (−1.32 to +0.35)	0.64	1.000	0.720
IABP at 24 h	1.11 (0.64 to 1.99)	0.71	1.000	0.720
Cardiac index at 24 h	−0.02 (−0.15 to +0.06)	0.72	1.000	0.720

CI, confidence interval; CRRT, continuous renal replacement therapy; FDR, false-discovery rate; IABP, intra-aortic balloon pump; ICU, intensive care unit; OR, odds ratio; PGD, primary graft dysfunction; RVFWS, right-ventricular free wall strain; VA-ECMO, venoarterial extracorporeal membrane oxygenation; VIS, vasoactive-inotropic score.

Exposure: each 5-unit *decrease* in the absolute magnitude of post-bypass RV free wall strain (i.e., a 5% less-negative RVFWS value). Effect estimates are adjusted for the pre-transplant weighted risk score. Outcomes are listed by ascending raw *p*-value within each tier (primary, then secondary). Bold values indicate statistical significance at the 0.05 threshold under that column's criterion.

a Bonferroni-adjusted *p*-value across 11 secondary outcomes (raw *p* × 11, capped at 1.000); significance threshold *p* < 0.05. The primary outcome was tested without multiple-comparisons adjustment.

b Benjamini–Hochberg FDR adjusted *q*-value across 11 secondary outcomes; significance threshold *q* < 0.05.

c Severe primary graft dysfunction was defined by adapting the 2014 ISHLT consensus criteria (29), as left-ventricular, right-ventricular, or biventricular graft dysfunction within 24 h of transplantation, requiring either high-dose inotropic support or mechanical circulatory support (VA-ECMO, IABP, or temporary ventricular assist device). PGD events include all severe subtypes; subtype-specific analyses were not performed because of small subtype event counts. This composite is broader than the 2014 consensus category of severe PGD-LV.

##### 2.5.1.2 Sample size and power

This study was a retrospective analysis of all consecutive eligible heart-transplant recipients during a defined twelve-month interval; no *a priori* power calculation was performed. With 14 primary-outcome events, multivariable analyses adjusted for the pre-transplant weighted risk score as a single composite covariate preserved an approximate events-per-variable ratio of ∼7, near the customary minimum threshold for logistic regression. Firth penalization was used to mitigate small-sample bias and address potential separation, and the use of a single composite adjustment covariate (rather than multiple individual baseline covariates) was deliberately chosen to minimize overfitting, given the limited event count. The study should, therefore, be interpreted as a hypothesis-generating, signal-finding analysis intended to motivate larger prospective studies rather than as a definitive estimate of effect size.

#### 2.5.2 Non-linear assessment and threshold exploration

Locally weighted scatterplot smoothing (LOESS) curves were generated to visually explore the association between RVFWS and outcomes. A potential inflection point was descriptively identified, where the estimated risk appeared to change with the RVFWS. The RVFWS was then dichotomized at the observed threshold to create a binary exposure variable for secondary analyses. Formal segmented (breakpoint) regression was considered but not pursued, as only 14 primary-outcome events, a breakpoint estimate, and its confidence interval would be unstable and could imply a precision that the data do not support. The −10% value is, therefore, presented as an exploratory, hypothesis-generating reference point identified by visual inspection of the LOESS fit rather than a statistically derived cut-point.

#### 2.5.3 Determinants of RVFWS

To identify pre-transplant factors associated with RVFWS, least absolute shrinkage and selection operator (LASSO) regression analysis was performed. The primary LASSO model treated RVFWS as a binary variable based on the identified threshold. For robustness, a secondary LASSO model treated RVFWS as a continuous outcome. Model selection was performed using cross-validation to determine the optimal penalty parameters.

#### 2.5.4 Model performance

Receiver operating characteristic (ROC) curve analysis was used to assess the discrimination of the LASSO model. Apparent performance was evaluated using bootstrap resampling to estimate the 95% CIs for the area under the curve (AUC). To assess the out-of-sample predictive performance, stratified 5-fold nested cross-validation was performed, with internal cross-validation used to select the optimal penalty parameter within each training fold.

To compare the prognostic performance of qualitative intraoperative RV function grading with quantitative RVFWS for 90-day mortality, separate multivariable Firth-corrected logistic regression models were constructed, each adjusted for the pre-transplant risk score. The predicted probabilities from each model were used to assess discrimination using the AUC with bootstrap-derived 95% CIs. Differences in the AUC between the correlated models were evaluated using DeLong's test, and the model accuracy was further assessed using the Brier score. To determine whether RVFWS provided incremental prognostic value beyond subjective grading, a combined model, including both qualitative RV function grade and RVFWS, was constructed. All performance analyses were considered exploratory, given the limited number of outcome events.

All tests were two-sided, and a *p*-value of <0.05 was considered statistically significant for the primary outcome.

## 3 Results

### 3.1 Cohort characteristics

A total of 147 isolated adult heart transplant recipients were included (Figure 1). The mean recipient age was 56.7 years (SD 11.7), and 33 recipients (22.4%) were female. The median post-bypass RVFWS was −13.9% (IQR −19.3 to −9.6). Fourteen recipients (9.5%) died within 90 days of transplantation. The detailed baseline characteristics and outcomes are presented in Tables 2, 3.

**Table 2:** Pre-transplant characteristics (*N* = 147).

Variable	Value
Recipient characteristics	
Age, years, mean (SD)	56.67 (11.67)
Female sex, *n* (%)	33 (22.4)
BMI, kg/m², mean (SD)	29.60 (4.60)
Diabetes, *n* (%)	53 (36.1)
Hypertension, *n* (%)	86 (58.5)
Pre-hospitalized, *n* (%)	70 (47.6)
Waitlist status 1, *n* (%)	9 (6.1)
Waitlist status 2, *n* (%)	47 (32.0)
Waitlist status 3, *n* (%)	36 (24.5)
Waitlist status 4, *n* (%)	25 (17.0)
Waitlist status 6, *n* (%)	30 (20.4)
Ischemic cardiomyopathy, *n* (%)	47 (32.0)
Pre-transplant LVAD, *n* (%)	44 (29.9)
Pre-transplant creatinine, mg/dL, mean (SD)	1.32 (0.51)
Prior sternotomy, *n* (%)	75 (51.0)
Donor characteristics	
Age, years, mean (SD)	39.44 (10.65)
Female sex, *n* (%)	58 (39.5)
BMI, kg/m², mean (SD)	27.60 (6.81)
PHM ratio, mean (SD)	0.91 (0.17)
Female-to-male mismatch, *n* (%)	35 (23.8)
Hypertension, *n* (%)	40 (27.2)
Diabetes, *n* (%)	7 (4.8)
LVEF, %, mean (SD)	60.64 (7.98)
Cause of death—CVA/ICH, *n* (%)	38 (25.9)
Cause of death—blunt trauma, *n* (%)	26 (17.7)
Cause of death—hypoxia, *n* (%)	49 (33.3)
Cause of death—other, *n* (%)	34 (23.1)
Donor distance, nautical miles, mean (SD)	464.96 (401.49)
DCD recovery, *n* (%)	71 (48.3)
Allograft ischemic time, minutes, mean (SD)	266.68 (86.68)
Total CPB duration, minutes, mean (SD)	153.46 (67.15)

BMI, body mass index; CPB, cardiopulmonary bypass; CVA/ICH, cerebrovascular accident/intracerebral hemorrhage; DCD, donation after circulatory death; LVAD, left-ventricular assist device; LVEF, left-ventricular ejection fraction; PHM, predicted heart mass; SD, standard deviation.

**Table 3:** Postoperative hemodynamic parameters, RV dysfunction, and clinical outcomes (*N* = 147).

Variable	Value
Post-bypass RV dysfunction grade, *n* (%)	
None	84 (57.1)
Mild	42 (28.6)
Moderate	19 (12.9)
Severe	2 (1.4)
Severe PGD, *n* (%)	10 (6.8)
VA-ECMO before ICU, *n* (%)	9 (6.1)
VA-ECMO after ICU, *n* (%)	7 (4.8)
IABP at 24 h, *n* (%)	7 (4.8)
VIS at 24 h, mean (SD)	10.95 (5.54)
VIS at 72 h, mean (SD)	7.64 (5.54)
Cardiac index at 24 h, L/min/m^2^, mean (SD)	3.07 (0.69)
Cardiac index at 72 h, L/min/m^2^, mean (SD)	3.11 (0.72)
CRRT, *n* (%)	42 (28.6)
ICU length of stay, days, mean (SD)	13.00 (11.85)
30-day mortality, *n* (%)	7 (4.8)
90-day mortality, *n* (%)	14 (9.5)

CRRT, continuous renal replacement therapy; ICU, intensive care unit; RV, right ventricle; SD, standard deviation; VA-ECMO, venoarterial extracorporeal membrane oxygenation; VIS, vasoactive-inotropic score.

### 3.2 Observer agreement for RVFWS

The inter-observer agreement for RVFWS between the attending anesthesiologists’ real-time measurements and the study observer's retrospective measurements was excellent (66 patients), with an intraclass correlation coefficient (ICC) (2,1) = 0.92 (95% CI 0.87–0.95; *p* < 0.001) (Supplementary Table S2). The intra-observer agreement for RVFWS based on repeated measurements by the study observer in 20 patients was also excellent, with ICC (2,1) = 0.96 (95% CI 0.91–0.98; *p* < 0.001) (Supplementary Table S3). Bland–Altman analysis confirmed minimal systematic bias between measurements, with a mean bias of +0.17% (95% limits of agreement, −5.30% to +5.64%) for the inter-observer comparison and −0.29% (−4.02% to +3.45%) for the intra-observer comparison (Supplementary Figures S1, S2).

### 3.3 RVFWS and postoperative outcomes

After adjustment for pre-transplant risk factors incorporated into the weighted risk score (donor age, donor diabetes, size mismatch, total ischemic time, female-to-male mismatch, pre-transplant LVAD use, and prior sternotomy), lower-magnitude (less negative) RVFWS was independently associated with higher 90-day mortality. Each 5% decrease in the absolute magnitude of RVFWS (i.e., a less negative value) was associated with an approximately threefold increase in the odds of 90-day mortality (OR 2.84, 95% CI 1.65–5.58; *p* < 0.001).

Among the eleven pre-specified secondary outcomes, lower-magnitude RVFWS was associated with severe primary graft dysfunction (OR 2.70, 95% CI 1.47–5.86; raw *p* = 0.001), 30-day mortality (OR 2.94, 95% CI 1.42–8.01; raw *p* = 0.002), need for postoperative CRRT (OR 1.58, 95% CI 1.18–2.16; raw *p* = 0.001), VIS at 24 h (median increase +0.76, 95% CI 0.00 to +1.28; raw *p* = 0.01), and VA-ECMO initiated before transfer to the ICU (OR 1.71, 95% CI 1.00–3.21; raw *p* = 0.04). After Bonferroni adjustment for eleven secondary comparisons (threshold *p* < 0.0045), the associations with severe PGD (Bonferroni-adjusted *p* = 0.011), CRRT (0.011), and 30-day mortality (0.022) remained statistically significant; the associations with VIS at 24 h (adjusted *p* = 0.110) and VA-ECMO before ICU (0.440) did not. Under the less-conservative Benjamini–Hochberg false-discovery-rate adjustment, the same three associations (severe PGD, CRRT, and 30-day mortality) plus VIS at 24 h remained significant (BH *q* = 0.006, 0.006, 0.007, and 0.028, respectively). The association with VA-ECMO initiated after ICU transfer did not reach statistical significance after any adjustment (OR 1.63, 95% CI 0.91–3.27; raw *p* = 0.10) (Table 1).

In sensitivity analyses, the association between lower-magnitude RVFWS and 90-day mortality was found to be robust. When DCD status was added directly to the primary multivariable model, it was not independently associated with 90-day mortality (OR 0.95, 95% CI 0.27–3.20; *p* = 0.94), and the RVFWS association remained essentially unchanged (OR 2.79, 95% CI 1.61–5.53; *p* < 0.001). Excluding the nine recipients who required intraoperative VA-ECMO, the association between RVFWS and 90-day mortality remained robust (OR 3.15, 95% CI 1.55–7.91; *p* < 0.001). Given the small number of excluded recipients and the limited secondary-outcome event counts, only the primary outcome was re-modeled in this restricted cohort.

Post-bypass RVFWS and post-transplant outcomes were compared between the 71 DCD and 76 DBD recipients (Supplementary Table S4). RVFWS distributions were similar between groups [median −14.6% (IQR −19.5 to −11.1) for DCD vs. −13.6% (IQR −18.6 to −7.7) for DBD; *p* = 0.12], and no outcome—including 90-day mortality [5/71 (7.0%) vs. 9/76 (11.8%); *p* = 0.40], severe PGD, post-transplant CRRT, vasoactive-inotropic score, cardiac index, or ICU length of stay—differed significantly by procurement type. Given the modest subgroup sizes, these comparisons were descriptive and not powered for confirmatory subgroup inference.

### 3.4 Non-linear assessment of RVFWS and determinants

LOESS curve visualization supported the regression findings and showed clustering of 90-day mortality events at RVFWS values less negative than approximately −10% (Figure 2). This threshold exploration was hypothesis-generating and was performed to simplify the subsequent predictive modeling.

**Figure 2:**
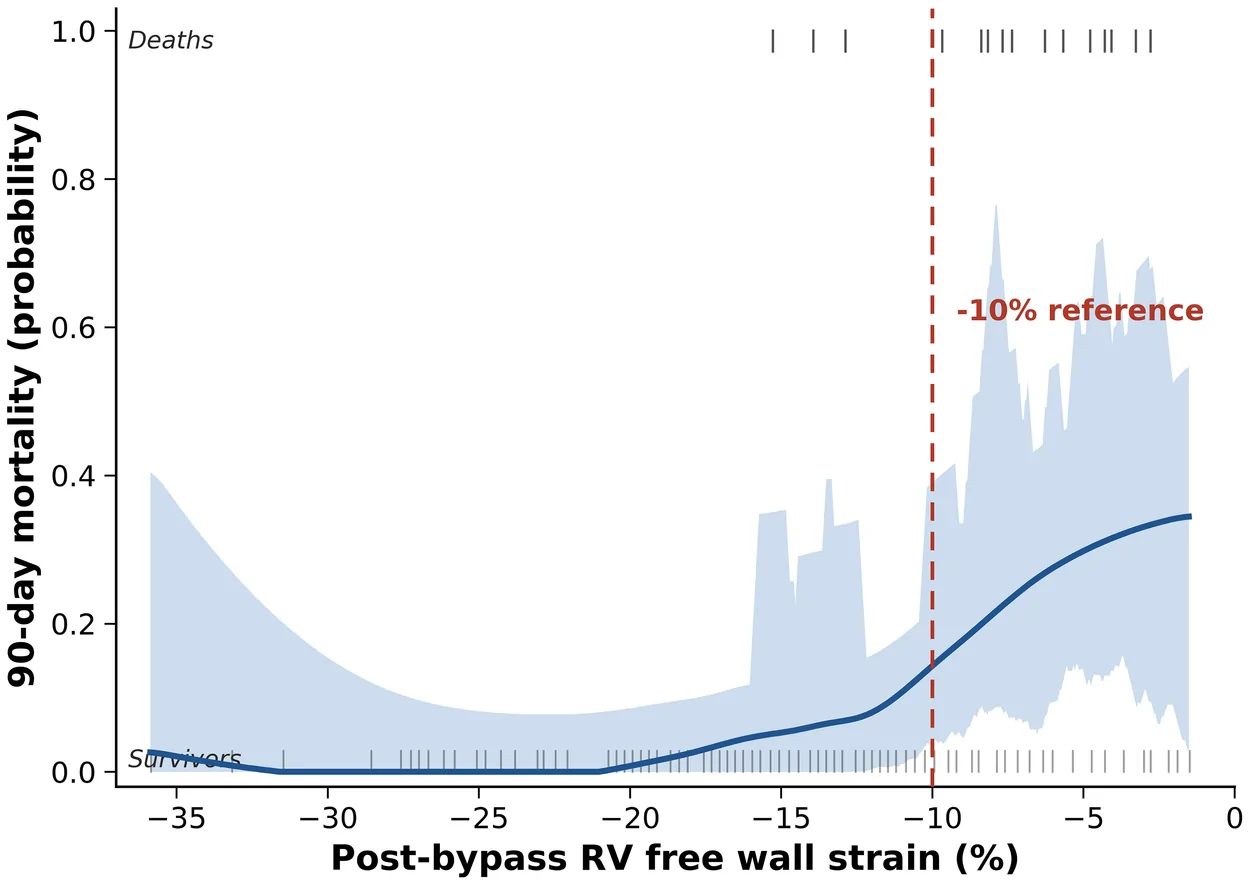
LOESS curve plotting 90-day mortality probability as a function of post-bypass RV free wall strain, showing that the majority of events cluster at RVFWS values less negative than approximately −10%. The shaded band represents the 95% confidence interval, the vertical dashed line marks the −10% reference value, and the rug plot along the axes indicates individual patients (deaths shown above, survivors below). LOESS, locally weighted scatterplot smoothing; RV, right ventricle; RVFWS, right-ventricular free wall strain.

A LASSO model constructed to identify pre-transplant determinants of RVFWS ≥−10% selected donor age, total ischemic time, donation after brain death, pre-transplant recipient creatinine, recipient BMI, and cardiopulmonary bypass duration as associated factors. The apparent (in-sample) discrimination of this model was good (AUC 0.79, 95% CI 0.70–0.87). However, stratified fivefold nested cross-validation demonstrated more modest out-of-sample discrimination (AUC 0.63, 95% CI 0.53–0.74), consistent with optimism in the apparent AUC and potential instability due to the limited sample size. In the continuous modeling of RVFWS, similar pre-transplant factors were identified, although donation after brain death and recipient creatinine levels were not consistently associated. The receiver operating characteristic curves for the apparent and cross-validated models are shown in Supplementary Figure S3.

### 3.5 Qualitative versus quantitative RV assessment

Qualitative intraoperative RV dysfunction grading demonstrated a stepwise association with quantitative RVFWS, with substantial within-category variability. The median RVFWS was −16.35% (IQR −20.00 to −10.90) among patients with no RV dysfunction, −13.15% (IQR −16.93 to −7.90) among those with mild dysfunction, and −8.70% (IQR −12.90 to −6.00) among those with moderate-to-severe dysfunction. Pairwise comparisons confirmed significant differences in RVFWS across categories (Wilcoxon rank-sum with Benjamini–Hochberg adjustment: none vs. mild *p* = 0.025; none vs. moderate/severe *p* = 0.001; mild vs. moderate/severe *p* = 0.036), supporting that RVFWS captures a graded spectrum of RV dysfunction underlying the subjective assessment (Figure 3).

**Figure 3:**
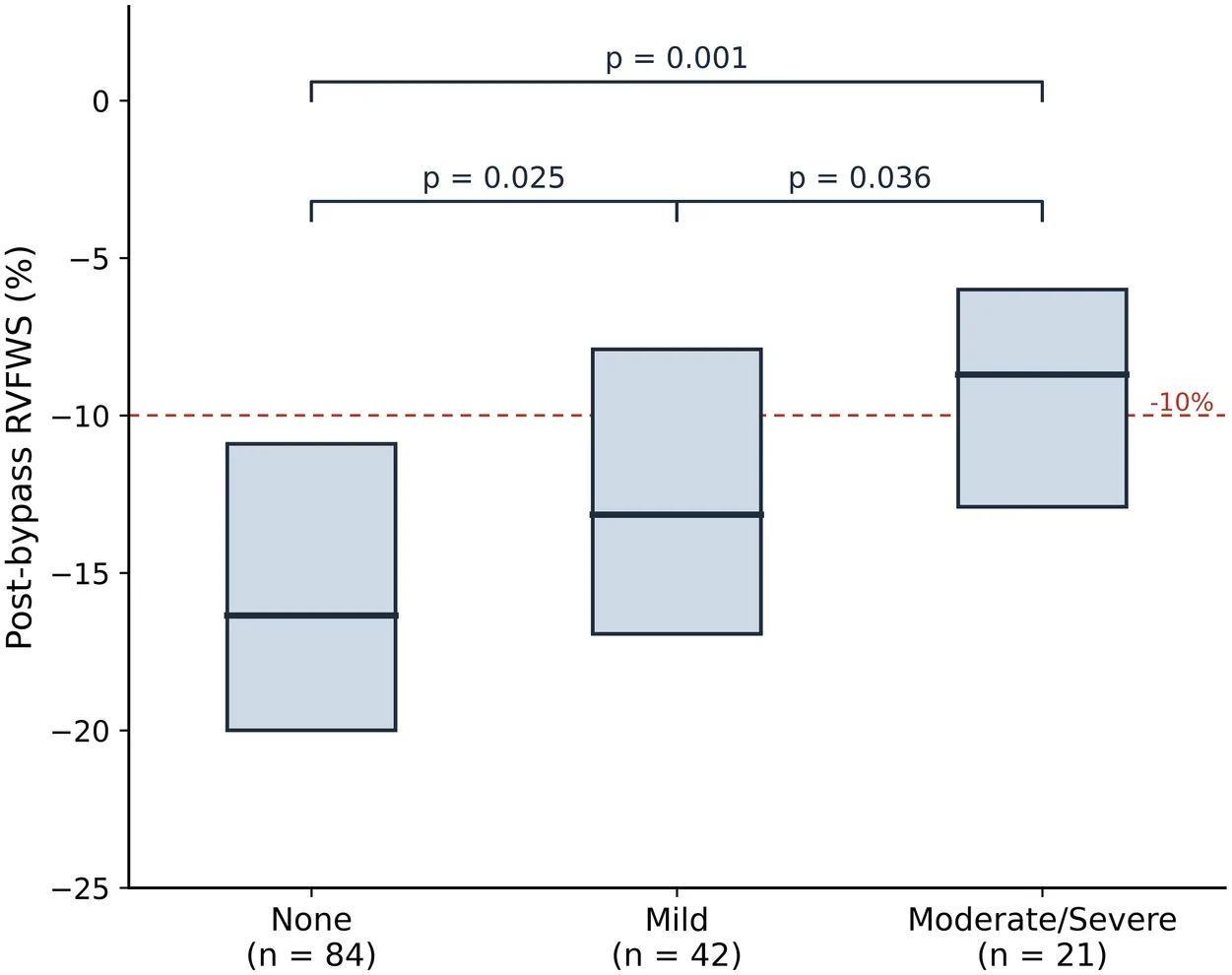
Distribution of post-bypass RVFWS across qualitative intraoperative RV dysfunction grades (none, *n* = 84; mild, *n* = 42; and moderate/severe, *n* = 21; moderate and severe combined owing to only two severe cases). Boxes indicate the interquartile range and the horizontal line the median; whiskers are not shown. Pairwise differences were assessed using the Wilcoxon rank-sum test with Benjamini–Hochberg adjustment. RVFWS, right-ventricular free wall strain; RV, right ventricle.

Model discrimination for 90-day mortality was higher when RV function was quantified using RVFWS than when it was graded qualitatively. The model incorporating RVFWS demonstrated good discrimination (AUC 0.83, 95% CI 0.73–0.91), whereas the model incorporating the qualitative RV dysfunction grade showed only modest discrimination (AUC 0.68, 95% CI 0.50–0.83). The DeLong test comparing the correlated AUCs did not reach statistical significance (*p* = 0.15), likely reflecting limited power given the low event count. Consistent with the improved predictive accuracy, the Brier score was lower for the RVFWS model (0.074) than for the qualitative-grade model (0.081). In a combined model, including both RVFWS and qualitative RV grading, RVFWS remained independently associated with 90-day mortality (*p* = 0.0004) while qualitative RV grading did not, suggesting that RVFWS captures prognostic information beyond subjective assessment. The receiver operating characteristic curves for both models are shown in Supplementary Figure S4.

## 4 Discussion

### 4.1 Principal findings

In this single-center cohort of 147 isolated adult heart transplant recipients, our primary hypothesis was supported: lower-magnitude (less negative) intraoperative post-bypass RVFWS was independently associated with higher 90-day mortality after adjustment for the pre-transplant risk score (OR 2.84 per 5% decrease in magnitude, 95% CI 1.65–5.58; *p* < 0.001). Our secondary hypotheses were only partially supported. Among the eleven pre-specified secondary outcomes, lower-magnitude RVFWS was associated with severe primary graft dysfunction, 30-day mortality, need for postoperative CRRT, higher VIS at 24 h, and VA-ECMO initiated before transfer to the ICU in the raw analysis. Critically, 3 of these associations (severe PGD, CRRT, and 30-day mortality) remained statistically significant under the most stringent Bonferroni correction across all 11 secondary outcomes, and 4 (the same 3 plus VIS at 24 h) remained significant under the less-conservative Benjamini–Hochberg false-discovery-rate adjustment. The association between RVFWS and post-bypass VA-ECMO did not retain significance under either correction. The RVFWS additionally outperformed qualitative intraoperative RV grading for predicting 90-day mortality in terms of AUC, Brier score, and in a combined multivariable model, consistent with our exploratory secondary aim. The persistence of the associations between mortality, PGD, and CRRT after stringent multiple-comparisons correction supports the interpretation that intraoperative post-bypass RVFWS captures clinically meaningful early allograft dysfunction rather than reflecting chance findings from multiple tests.

### 4.2 RVFWS in the broader cardiac surgery and transplantation literature

Assessment of the RV is a long-standing clinical challenge, particularly intraoperatively and especially in heart-transplant patients. Complex myocardial geometry, motion, and rotation within the mediastinum complicate TEE-based RV assessment. In addition, post-transplant evaluation must consider the effects of donor heart quality, ischemia–reperfusion injury, loading conditions, and stress related to cardiopulmonary bypass. Speckle-tracking RV strain has been proposed as an alternative that directly quantifies RV function compared with conventional measures such as TDI S’, TAPSE, fractional area change, and subjective assessment (23). In several RV disease states, RV free wall strain has shown strong prognostic value and compares favorably with conventional RV parameters in predicting adverse outcomes (24, 25). In the heart-transplant population specifically, RV strain measured on postoperative follow-up has been shown to predict graft-related RV dysfunction (26, 27).

### 4.3 Contribution of this study

To the best of our knowledge, this is the first study to evaluate intraoperative RV strain in adult heart transplantation. Our finding that post-bypass RVFWS is independently associated with early mortality and other postoperative adverse events extends prior outpatient and post-discharge data into the immediate intraoperative window when management decisions (mechanical support, inotrope titration, and delayed chest closure) are still actionable. Consistent with prior post-discharge work, RVFWS captured graft dysfunction not fully explained by donor and recipient pre-transplant characteristics and showed higher discrimination than qualitative intraoperative RV grading on AUC, Brier score, and in a combined multivariable model, although the difference in AUC did not reach statistical significance. Although qualitative grading and RVFWS were correlated in a stepwise manner, the substantial within-category variability suggests that subjective grading alone may obscure prognostically meaningful gradations of RV function.

Several donor, recipient, and operative factors—including donor age, ischemic time, recipient creatinine level, body mass index, and cardiopulmonary bypass duration—have been associated with mortality after heart transplantation in prior studies. Because these variables reflect baseline graft quality, recipient vulnerability, and operative stress, we hypothesized that they might also be associated with early post-implant RV function. LASSO regression was, therefore, used to explore the ability of these pre-transplant and perioperative characteristics to predict early RV dysfunction, as measured by RVFWS. Although apparent discrimination was good, the cross-validated performance was modest, highlighting the limited ability of static baseline characteristics alone to reliably identify patients who develop early RV dysfunction. In contrast, RVFWS was strongly associated with early clinical outcomes, suggesting that RV strain captures dynamic perioperative injury processes that are not fully explained by pre-transplant risk profiles.

Exploratory LOESS analysis suggested clustering of adverse events once the strain became less negative than approximately −10%. This inflection point was used to contextualize risk patterns rather than to define a strict clinical cutoff value. It should, therefore, be viewed as a hypothesis-generating signal rather than an actionable clinical threshold and requires prospective validation before it can inform management.

Right-ventricular myocardial work (RVMW) has recently been proposed as a complementary and less load-dependent measure of RV performance. Strain is influenced by the loading conditions, whereas non-invasive pressure–strain loop analysis incorporates an estimate of the afterload alongside deformation. This may allow for better separation of intrinsic contractility from load than strain alone. Landra et al. applied non-invasive RV pressure–strain loop analysis on preoperative transthoracic echocardiography in candidates for durable left-ventricular assist device implantation and found that RVMW indices predicted early right heart failure and long-term mortality (30). However, it is unknown whether this advantage extends to the intraoperative transesophageal setting. Post-bypass loading conditions change rapidly in heart transplantation, and non-invasive estimation of RV systolic pressure from the tricuspid regurgitation jet is frequently unreliable immediately after separation from bypass. Moreover, the pressure–strain loop software has not been validated for transesophageal acquisition. Direct pulmonary artery pressure measurements are, however, routinely available in this population. Invasively calibrated intraoperative RVMW is, therefore, a feasible and attractive target for prospective studies alongside RVFWS.

### 4.4 Limitations

This study had several limitations. As a retrospective single-center analysis of a high-volume program with an active DCD heart-transplant pathway, the generalizability to centers with differing donor-selection criteria, surgical techniques, preservation strategies, and perioperative management is limited. The sample size and event count for the primary outcome were modest (14 90-day deaths in 147 recipients), and the study was not powered by an *a priori* calculation. The analysis was framed as a hypothesis-generating signal-finding effort to motivate prospective work, and Firth penalization combined with a single composite pre-transplant risk score adjustment was used to minimize small-sample bias and overfitting. However, the limited event count restricts our power to detect smaller effects, as illustrated by the VA-ECMO associations, which did not reach significance after adjustment for multiple comparisons despite biologically plausible point estimates. The proportion of recipients transplanted via a DCD pathway (48.3%) was substantially higher than that at most contemporary programs and reflected an institution-specific donor pathway, further limiting generalizability to centers with different donor selection and preservation practices. Given the limited event count, we did not attempt a fully specified multivariable model that incorporated individual donor, recipient, and operative covariates. With 14 primary outcome events, such a model would be substantially overfitted. A univariable screen followed by an expanded multivariable analysis is better suited to the larger prospective multicenter cohort required to confirm these findings.

The RVFWS was measured at a single post-bypass time point. To minimize variability, the last adequate end-of-case image (after chest closure, with final inotropic support, and volume status intended to be at a stabilized end-of-case state) was selected for each patient; however, residual variability in loading conditions, ongoing low-dose inotrope titration, and time from closure to image acquisition remain inherent limitations of an end-of-case single-time-point measurement. Strain measurements, including RVFWS, are affected by electrical activation patterns, including epicardial pacing (28). Post-transplant pacing patterns at our institution include atrial pacing, atrioventricular pacing, and no pacing. Although pacing was documented during the case, it could not be reliably time-locked to the specific loop used for offline strain measurement and was therefore not evaluated as a covariate in this analysis. Unrecorded pacing state at the instant of measurement was retained as an explicitly acknowledged potential confounder of post-bypass RVFWS. The image quality of the post-transplant RV is variable, and speckle tracking depends on adequate 2D imaging. Inter- and intra-observer agreement for RVFWS was excellent by intraclass correlation, and Bland–Altman analysis showed minimal systematic bias between observers. However, the limits of agreement (up to approximately ±5.5% for inter-observer measurements) indicate that individual readings near the −10% reference can occasionally differ enough to fall on either side of it, underscoring the need for cautious individual-level interpretation and prospective confirmation.

Qualitative versus quantitative comparisons should be interpreted with caution. Qualitative intraoperative RV grading was performed by the attending cardiac anesthesiologist without a formal inter-rater reliability assessment, and the low absolute event count for 90-day mortality (*n* = 14) limited the power of the DeLong test (which did not reach statistical significance) even when the absolute discrimination gap was substantial (AUC 0.83 vs. 0.68). The consistency of incremental prognostic value of the RVFWS across the combined-model and Brier-score analyses suggests a real signal that warrants prospective confirmation. The proportion of recipients placed on intraoperative VA-ECMO in this cohort (9 of 147; 6.1%) was lower than reported at some other centers, which may reflect differences in institutional protocols for early mechanical support. Because RVFWS was acquired before ECMO cannulation in these patients, the strain values reported here were not directly affected by ECMO unloading, but centers with higher intraoperative ECMO utilization or post-ECMO measurement may not see comparable effect sizes. Finally, LASSO modeling was used to identify factors associated with early RV dysfunction rather than to establish causal relationships, and despite cross-validation, unmeasured confounding factors cannot be excluded.

Despite these limitations, our data provide novel evidence that intraoperative post-bypass RVFWS is feasible to obtain in routine heart-transplant practice, captures information not provided by qualitative grading, and is independently associated with clinically meaningful early postoperative outcomes, including outcomes (90-day mortality, severe PGD, CRRT, and 30-day mortality) that survive stringent multiple-comparisons correction. These findings support the potential role of RVFWS as an actionable intraoperative marker of early allograft dysfunction and motivate prospective, multicenter validation.

## 5 Conclusions

In this single-center retrospective cohort study of 147 adult heart-transplant recipients, lower-magnitude intraoperative post-bypass RVFWS was independently associated with higher 90-day mortality and other early adverse postoperative outcomes after adjustment for *a priori* pre-transplant risk score, and it outperformed qualitative intraoperative RV grading. The RVFWS appears to be feasible for incorporation into routine intraoperative TEE assessments during heart transplantation. Prospective, adequately powered, multicenter validation, including pre-specified cut-points and integration into perioperative decision-making, is needed before routine clinical adoption.

## Data Availability

The de-identified data supporting the conclusions of this article will be made available by the authors to qualified investigators upon reasonable request and following review by the Vanderbilt University Medical Center Institutional Review Board. Code used for the statistical analyses is available from the corresponding author upon request.
