# Supplementary material for: Use of intraoperative right ventricular strain as a predictor of outcomes in adult heart transplant patients
Source: Front Cardiovasc Med. 2026 Oct 7;13:1890033. doi: 10.3389/fcvm.2026.1890033 (PMC13683753; doi:10.3389/fcvm.2026.1890033)
Supplement: Supplementary file 1 [file Datasheet1.docx]

**Supplementary Material**

**Manuscript:** Use of Intraoperative Right Ventricular Strain as a Predictor of Outcomes in Adult Heart Transplant Patients

**Journal:** *Frontiers in Cardiovascular Medicine* — Cardiovascular Imaging section

**Corresponding author:** John Bryant, MD (john.bryant@vumc.org)

**Contents**

Supplementary Table S1. Pre-transplant Risk Score

Supplementary Table S2. Inter-observer Variability for Post-bypass RVFWS

Supplementary Table S3. Intra-observer Variability for Post-bypass RVFWS

Supplementary Table S4. Post-transplant RVFWS and Outcomes by Procurement Type (DCD vs. DBD)

Supplementary Figure S1. Inter-observer Agreement for Post-bypass RVFWS (Bland–Altman)

Supplementary Figure S2. Intra-observer Agreement for Post-bypass RVFWS (Bland–Altman)

Supplementary Figure S3. ROC Curves for the LASSO Model Predicting Worsening Post-bypass RVFWS

Supplementary Figure S4. ROC Curves Comparing Quantitative RVFWS with Qualitative RV Grading for 90-day Mortality

**Supplementary Table S1. Pre-transplant Risk Score**

The weighted pre-transplant risk score was used solely for multivariable adjustment in the present analysis. It was developed a priori — prior to analysis of the present cohort — using our institution’s heart-transplant dataset through December 2024 (n ≈ 600), with severe primary graft dysfunction and/or early mortality as the outcome. The present study cohort (n = 147, transplanted January–December 2025) is entirely subsequent to the derivation period; there is therefore no patient overlap between the derivation dataset and the analytic cohort, and adjustment for the score does not constitute circular adjustment. Ridge regression was used to mitigate multicollinearity among correlated predictors. To convert coefficients into a usable score, ridge-regression coefficients (β) were exponentiated (e^β^) to yield relative-risk-scaled weights, which were proportionally rescaled and rounded to the nearest half-integer to generate the additive point values shown below; a patient’s total score is the sum of the points for their present risk factors. Candidate variables were selected a priori from prior literature and clinical relevance rather than by data-driven selection in the present cohort. The score was developed solely for adjustment within this study, has not undergone external validation, and is not intended as a clinical decision-making tool.

| Variable | Score |
| --- | --- |
| Donor age >35 years to 50 years | 1.5 |
| Donor age >50 years | 3 |
| Female-to-male donor–recipient mismatch | 1 |
| Diabetic donor | 2 |
| Donor undersized by PHM ratio <0.8 | 1.5 |
| Total ischemic time >4 hours and <5 hours | 3 |
| Total ischemic time >5 hours | 5 |
| Pre-transplant LVAD | 4 |
| Prior sternotomy that was not an LVAD implant | 2 |

LVAD, left ventricular assist device; PHM, predicted heart mass.

**Supplementary Table S2. Inter-observer Variability for Post-bypass RVFWS**

Paired post-bypass RVFWS measurements in 66 patients: the study observer’s offline post-processing measurement (left) and the intraoperative real-time measurement performed by a separate attending anesthesiologist (right). Intraclass correlation coefficient (ICC) (2,1) = 0.92 (95% CI 0.87–0.95; *p* < 0.001). Bland–Altman analysis showed minimal systematic bias (mean bias +0.17%; 95% limits of agreement −5.30% to +5.64%); see Supplementary Figure S1.

| Patient ID | Study measurement (RVFWS, %) | Operating-room measurement (RVFWS, %) |
| --- | --- | --- |
| 1 | −14.4 | −12.6 |
| 2 | −17.1 | −19.5 |
| 3 | −14.1 | −12.9 |
| 4 | −15.7 | −15.5 |
| 6 | −26.7 | −29.6 |
| 8 | −7.9 | −11.1 |
| 9 | −18.3 | −18.9 |
| 10 | −29.8 | −27.0 |
| 12 | −15.4 | −14.2 |
| 13 | −15.9 | −16.3 |
| 16 | −20.1 | −17.3 |
| 17 | −27.1 | −24.3 |
| 20 | −17.0 | −16.0 |
| 22 | −13.0 | −17.1 |
| 24 | −13.6 | −14.4 |
| 27 | −4.3 | −11.6 |
| 29 | −13.7 | −12.9 |
| 31 | −35.9 | −39.8 |
| 34 | −9.2 | −9.5 |
| 35 | −11.7 | −10.2 |
| 36 | −2.0 | −6.4 |
| 38 | −19.5 | −17.6 |
| 42 | −12.1 | −15.8 |
| 44 | −6.0 | −9.6 |
| 49 | −15.3 | −13.4 |
| 52 | −11.5 | −11.2 |
| 55 | −27.6 | −30.0 |
| 56 | −2.2 | −4.7 |
| 57 | −10.3 | −15.4 |
| 60 | −17.0 | −19.5 |
| 62 | −12.6 | −10.9 |
| 65 | −8.7 | −8.5 |
| 66 | −15.3 | −11.0 |
| 67 | −16.4 | −15.1 |
| 74 | −14.5 | −10.3 |
| 75 | −19.4 | −18.0 |
| 76 | −11.6 | −12.1 |
| 78 | −11.2 | −14.2 |
| 79 | −20.0 | −26.2 |
| 81 | −7.7 | −6.3 |
| 84 | −20.3 | −20.7 |
| 87 | −16.0 | −19.9 |
| 88 | −6.8 | −6.0 |
| 90 | −24.2 | −18.4 |
| 91 | −10.2 | −11.1 |
| 93 | −13.3 | −14.0 |
| 96 | −11.8 | −13.8 |
| 97 | −17.6 | −13.6 |
| 100 | −20.0 | −20.7 |
| 101 | −4.8 | −4.5 |
| 104 | −16.7 | −18.2 |
| 107 | −14.5 | −10.3 |
| 114 | −10.0 | −11.7 |
| 115 | −15.1 | −16.7 |
| 118 | −20.4 | −20.4 |
| 120 | −13.0 | −14.0 |
| 124 | −10.0 | −8.4 |
| 125 | −22.5 | −25.7 |
| 126 | −33.2 | −30.2 |
| 127 | −27.3 | −26.2 |
| 136 | −19.1 | −14.8 |
| 144 | −13.8 | −16.1 |
| 169 | −16.3 | −12.5 |
| 173 | −10.6 | −10.4 |
| 183 | −18.5 | −17.6 |
| 188 | −26.2 | −22.4 |

ICC, intraclass correlation coefficient; RVFWS, right ventricular free wall strain.

**Supplementary Table S3. Intra-observer Variability for Post-bypass RVFWS**

Paired post-bypass RVFWS measurements in 20 patients: the original study measurement (left) and a repeated post-processing measurement by the same study observer (right). Intraclass correlation coefficient (ICC) (2,1) = 0.96 (95% CI 0.91–0.98; *p* < 0.001). Bland–Altman analysis showed minimal systematic bias (mean bias −0.29%; 95% limits of agreement −4.02% to +3.45%); see Supplementary Figure S2.

| Patient ID | Study measurement (RVFWS, %) | Repeated measurement (RVFWS, %) |
| --- | --- | --- |
| 4 | −15.7 | −14.6 |
| 5 | −3.7 | −2.9 |
| 9 | −18.3 | −16.7 |
| 25 | −19.8 | −23.8 |
| 34 | −9.2 | −9.7 |
| 87 | −16.0 | −14.7 |
| 89 | −17.5 | −14.1 |
| 91 | −10.2 | −12.2 |
| 114 | −10.0 | −11.7 |
| 115 | −15.1 | −16.7 |
| 118 | −20.4 | −20.4 |
| 130 | −20.6 | −19.5 |
| 140 | −7.7 | −7.6 |
| 142 | −3.0 | −4.9 |
| 144 | −13.8 | −15.2 |
| 148 | −5.7 | −3.8 |
| 152 | −25.8 | −25.1 |
| 156 | −25.1 | −23.4 |
| 158 | −19.6 | −16.6 |
| 162 | −6.3 | −4.2 |

ICC, intraclass correlation coefficient; RVFWS, right ventricular free wall strain.

**Supplementary Table S4. Post-transplant RVFWS and Outcomes by Procurement Type (DCD vs. DBD)**

Comparison of post-bypass right ventricular free wall strain (RVFWS) and post-transplant outcomes between recipients of donation-after-circulatory-death (DCD, n = 71) and donation-after-brain-death (DBD, n = 76) allografts. Fisher’s exact test was used for binary outcomes and the Wilcoxon rank-sum test for continuous outcomes. Comparisons are descriptive; given the modest subgroup sizes and the limited number of primary-outcome events, the study was not powered for formal donor-type effect-modification testing.

| Variable | DCD (n = 71) | DBD (n = 76) | p value |
| --- | --- | --- | --- |
| RVFWS, %, median (IQR) | −14.6 (−19.5 to −11.1) | −13.6 (−18.6 to −7.7) | 0.12 |
| Severe PGD, n (%) | 4 (5.6) | 6 (7.9) | 0.74 |
| Post-transplant CRRT, n (%) | 20 (28.2) | 22 (28.9) | >0.99 |
| 90-day mortality, n (%) | 5 (7.0) | 9 (11.8) | 0.40 |
| IABP at 24 h, n (%) | 4 (5.6) | 3 (3.9) | 0.71 |
| VIS at 24 h, median (IQR) | 9.88 (7.78–14.45) | 10.00 (7.00–13.22) | 0.71 |
| VIS at 72 h, median (IQR) | 6.07 (5.00–10.00) | 5.15 (3.94–10.00) | 0.28 |
| Cardiac index at 24 h, median (IQR) | 3.02 (2.60–3.55) | 2.98 (2.59–3.44) | 0.48 |
| Cardiac index at 72 h, median (IQR) | 3.00 (2.50–3.48) | 3.09 (2.80–3.38) | 0.31 |
| ICU length of stay, days, median (IQR) | 9.00 (5.25–16.00) | 9.00 (5.00–16.00) | 0.59 |

CRRT, continuous renal replacement therapy; DBD, donation after brain death; DCD, donation after circulatory death; IABP, intra-aortic balloon pump; ICU, intensive care unit; IQR, interquartile range; PGD, primary graft dysfunction; RVFWS, right ventricular free wall strain; VIS, vasoactive-inotropic score.

**Supplementary Figure S1. Inter-observer agreement for post-bypass RVFWS (Bland–Altman plot).**

Differences between the two observers’ paired post-bypass RVFWS measurements (n = 66) are plotted against their mean. The solid horizontal line indicates the mean bias (+0.17%); the dashed horizontal lines indicate the 95% limits of agreement (−5.30% to +5.64%); the vertical dashed line marks the −10% reference value. RVFWS, right ventricular free wall strain; SD, standard deviation.

**Supplementary Figure S2. Intra-observer agreement for post-bypass RVFWS (Bland–Altman plot).**

Differences between the original and repeated post-bypass RVFWS measurements by the same observer (n = 20) are plotted against their mean. The solid horizontal line indicates the mean bias (−0.29%); the dashed horizontal lines indicate the 95% limits of agreement (−4.02% to +3.45%); the vertical dashed line marks the −10% reference value. RVFWS, right ventricular free wall strain; SD, standard deviation.

**Supplementary Figure S3. Receiver operating characteristic (ROC) curves for the LASSO model predicting worsening post-bypass RVFWS (RVFWS ≥ −10%).**

Receiver operating characteristic (ROC) curves for the least absolute shrinkage and selection operator (LASSO) model predicting worsening right ventricular free wall strain (RVFWS ≥ −10%). The blue curve shows apparent (in-sample) discrimination (AUC 0.79, 95% CI 0.70–0.87); the red curve shows out-of-sample discrimination from stratified 5-fold nested cross-validation (AUC 0.63, 95% CI 0.53–0.74). Shaded bands represent bootstrap-derived 95% confidence intervals for sensitivity at each specificity (2,000 resamples). The dashed diagonal line represents the line of no discrimination. AUC, area under the curve; CI, confidence interval; LASSO, least absolute shrinkage and selection operator; RVFWS, right ventricular free wall strain.

**Supplementary Figure S4. Receiver operating characteristic (ROC) curves comparing quantitative RVFWS with qualitative RV grading for 90-day mortality.**

Receiver operating characteristic (ROC) curves comparing discrimination for 90-day mortality between the quantitative right ventricular free wall strain (RVFWS)–based model (blue) and the qualitative intraoperative RV function grading model (red), each adjusted for the pre-transplant risk score. The RVFWS model demonstrated an AUC of 0.83 (95% CI 0.73–0.91) versus 0.68 (95% CI 0.50–0.83) for the qualitative grading model (DeLong p = 0.15). Shaded bands represent bootstrap-derived 95% confidence intervals for sensitivity at each specificity (2,000 resamples). The dashed diagonal line represents the line of no discrimination. AUC, area under the curve; CI, confidence interval; RV, right ventricle; RVFWS, right ventricular free wall strain.
